# Identification of Cables1 as a critical host factor that promotes ALV-J replication *via* genome-wide CRISPR/Cas9 gene knockout screening

**DOI:** 10.1016/j.jbc.2024.107804

**Published:** 2024-09-21

**Authors:** Peng Liu, Jinghua Jiang, Yuntong Chen, Fei Gao, Suyan Wang, Mengmeng Yu, Yongzhen Liu, Ru Guo, Li Zhang, Zhuangzhuang Xu, Caiying Wang, Xiaole Qi, Yanping Zhang, Hongyu Cui, Yulu Duan, Sen Wu, Yulong Gao

**Affiliations:** 1 State Key Laboratory for Animal Disease Control and Prevention, Avian Immunosuppressive Diseases Division, Harbin Veterinary Research Institute, The Chinese Academy of Agricultural Sciences, Harbin, PR China; 2 State Key Laboratory of Animal Biotech Breeding, College of Biological Sciences, China Agricultural University, Beijing, PR China; 3 Frontiers Science Center for Molecular Design Breeding, China Agricultural University, Beijing, China; 4 Jiangsu Co-Innovation Center for the Prevention and Control of Important Animal Infectious Disease and Zoonose, Yangzhou University, Yangzhou, PR China; 5 National Poultry Laboratory Animal Resource Center, Harbin, PR China

**Keywords:** subgroup J avian leukosis virus, CRISPR screen, Cables1, p15, ubiquitination

## Abstract

Avian leukosis virus subgroup J (ALV-J), a member of the genus *Alpharetrovirus*, possesses a small genome and exploits a vast array of host factors during its replication cycle. To identify host factors required for ALV-J replication and potentially guide the development of key therapeutic targets for ALV-J prevention, we employed a chicken genome-wide CRISPR/Cas9 knockout library to screen host factors involved in ALV-J infection within DF-1 cells. This screening revealed 42 host factors critical for ALV-J infection. Subsequent knockout assays showed that the absence of the genes encoding cycle-regulatory proteins, namely, *Cables1*, *CDK1*, and *DHFR*, significantly inhibited ALV-J replication. Notably, *Cables1* knockout cell lines displayed the most pronounced inhibitory effect. Conversely, overexpression assays confirmed that Cables1 significantly promotes ALV-J replication. Immunoprecipitation assays further indicated that Cables1 specifically interacts with the viral protein p15 (viral protease) among all ALV-J proteins, enhancing ALV-J p15 polyubiquitination. Additionally, we identified 26 lysine residues of ALV-J p15 as key sites for ubiquitination, and their replacement with arginine attenuated the replication ability of ALV-J in both *in vitro* and *in vivo* assays. This study demonstrates that Cables1 is a critical replication-dependent host factor of ALV-J by enhancing p15 ubiquitination and thereby promoting viral replication. Overall, these findings contribute to a deeper understanding of the ALJ-V replication mechanism and offer a potential target for the prevention and control of ALV-J infection.

Avian leukosis virus (ALV) is an avian oncogenic retrovirus belonging to the *Alpharetrovirus* genus of the Retroviridae family. Phylogenetic analyses of the *env* gene, host range, and serological neutralization tests classify ALVs into 11 subgroups (ALV-A to ALV-K) (1, 2, 3). In contrast to other subgroups of ALV, ALV-J exhibits high pathogenicity (4, 5) and a broad host range encompassing commercial and non-commercial chickens, and wild birds (6, 7, 8). Moreover, infection with ALV-J leads to a significant reduction in egg production and high mortality rates in chickens (8). The widespread dissemination and evolution of ALV-J pose a significant threat to the poultry industry, causing substantial economic losses in China. However, no effective antiviral drugs or vaccines are available to combat ALV-J infection.

ALV-J, a single-stranded RNA virus, possesses three open reading frames encoding *gag*, *pol*, and *env* genes, flanked by 5ʹ and 3ʹ untranslated regions, and a poly-A tail (9). The *gag* gene encodes major structural components, including the capsid protein (p27), matrix protein (p19), p10, nucleocapsid protein (p12), and protease (p15) (10). The *pol* gene encodes reverse transcriptase (RT) and integrase (IN), which are necessary for the early phase of replication (10). The *env* gene encodes the surface (gp85) and transmembrane (gp37) proteins, critical for viral attachment and fusion with host cells (11, 12). While ALV-J encodes proteins required for particle formation, replication, and spread, similar to other retroviruses, it relies heavily on several host factors to complete its life cycle. For example, ribonucleoside-diphosphate reductase subunit M2 promotes ALV-J replication by interacting with p27 and activating the Wnt/β-catenin pathway in DF-1 cells (13). Moreover, the chicken Na^+^/H^+^ exchanger type 1 is required for ALV-J attachment to host cells (14). Therefore, a comprehensive understanding of the interaction mechanisms between host factors and ALV-J is crucial for elucidating viral replication and developing potential therapeutic targets to control the spread of ALV-J.

In recent years, various genetic screening methods, including transposon mutagenesis, RNA interference, complementary DNA (cDNA) overexpression, and genome-wide CRISPR/Cas9 screening, have been used to identify host factors associated with viral infections (15). Among these methods, genome-wide CRISPR/Cas9 screening offers high concordance in reproducibility and accuracy. This technique has successfully identified critical host dependency factors for viruses such as influenza A virus (16, 17), severe acute respiratory syndrome coronavirus 2 (18, 19), and porcine reproductive and respiratory syndrome virus (20, 21, 22).

Therefore, in this study, we utilized a chicken genome-wide CRISPR/Cas9 knockout (ChGeCKO) library to screen and identify host factors essential for ALV-J infection in immortal chicken embryo fibroblast (DF-1) cells. Following three rounds of screening, 42 candidate host factors were identified. Among these candidates, Cdk5 and Abl enzyme substrate 1 (Cables1), which acts as an adaptor molecule linking Abl and Cdk5 (23), emerged as a critical host factor for ALV-J infection, as confirmed through knockout and overexpression assays. Furthermore, our findings revealed that Cables1 interacts with p15, promoting its ubiquitination *via* the Lys26 site. This ubiquitination is essential for ALV-J replication in both *in vivo* and *in vitro* models. Our results demonstrate that chicken Cables1 is an important host factor in ALV-J replication.

## Results

### Host factors associated with ALV-J infection were identified using a chicken genome-wide CRISPR/Cas9 library (ChGeCKO)

**Figure 1:**
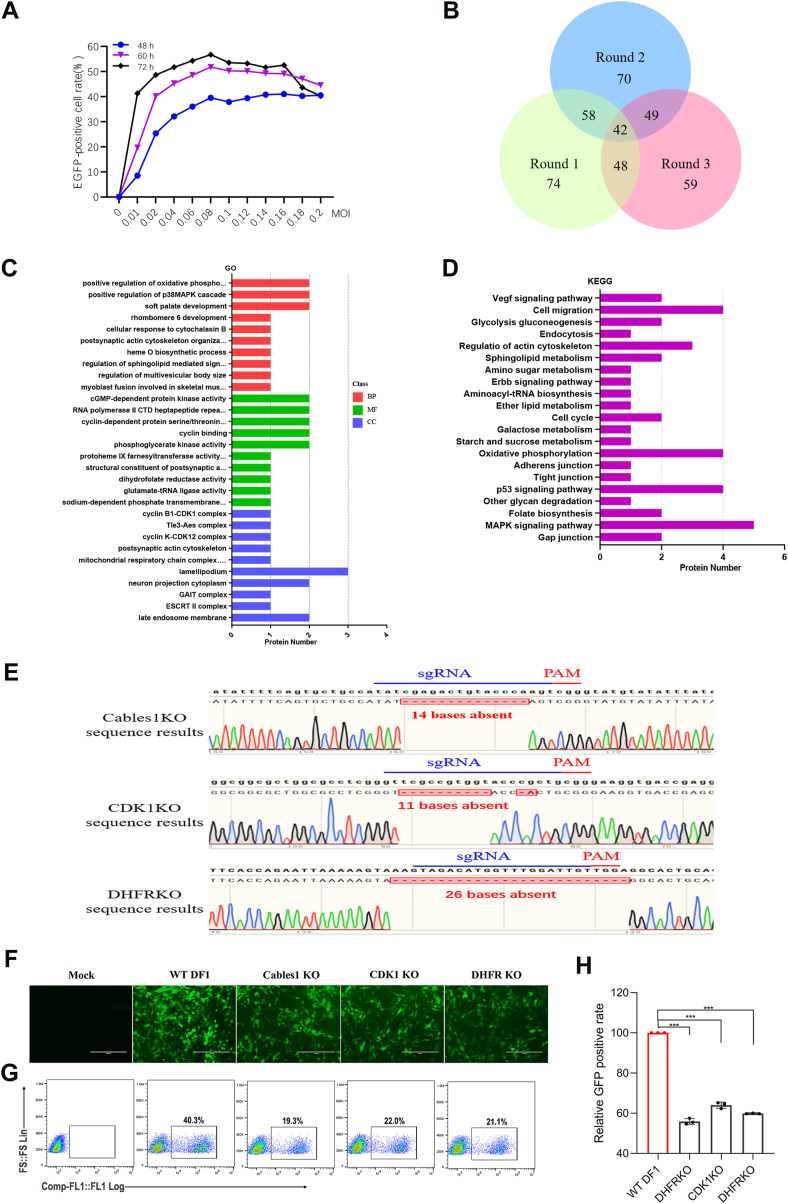
**Genome-wide genetic screening identifies host factors required for ALV-J infection.***A*, identification of the infection efficiency of ALV-J reporter virus RCAS(J)GFP in ChGeCKO cells at MOI of 0.01, 0.02, 0.04, 0.06, 0.08, 0.1, 0.12, 0.14, 0.016, 0.018 and 0.2. After 48, 60, and 72 h following infection, the infection efficiency was analyzed using a flow cytometer. *B*, venn diagrams indicate the overlapping enrichment genes from the three rounds of screening of host factors associated with ALV-J replication using the ChGeCKO library. In the initial, second, and third rounds of screening, there were 222, 218, and 207 enriched genes, respectively. Genes listed in the middle were identified in three screens. *C*, gene ontology analysis of the identical 42 candidate host genes from three ALV-J challenge rounds. *D*, KEGG pathway enrichment analyses of the identical 42 candidate host genes from three ALV-J challenge rounds. *E*, sequence analysis of the *Cables1*, *CDK1* and *DHFR* knockout DF-1 cells lines. *F*-*H*, *DHFR*, *CDK1*, and *Cables1* are essential genes for ALV-J infection. To detect the susceptibility of host factors to ALV-J, *DHFR*, *CDK1*, and *Cables1* knockout cell lines were inoculated with RCAS(J)GFP. *F*, after infection, the cells infected with RCAS(J)GFP were observed by fluorescence microscope. Scale bar: 200 μm. *G*, flow cytometer assays were conducted to determine the infection rate of RCAS(J)GFP based on positive cells. *H*, relative infection rates of RCAS(J)GFP were normalized to those of the WT DF-1 group. A representative experiment out of three repeats is shown, and data are shown as means ± SD for triplicates from the representative experiment. ∗, *p* < 0.05; ∗∗, *p* < 0.01; ∗∗∗, *p* < 0.001; ns, not significant.

To identify host factors required for ALV-J infection, we first optimized the infection efficiency of an ALV-J reporter virus in a ChGeCKO. This reporter virus, a replication-competent avian leucosis sarcoma virus (RCAS) (J) with enhanced GFP (EGFP) (RCAS(J)GFP), was used to infect ChGeCKO cells at various multiplicities of infection (MOI). We observed the highest infection efficiency (56.7%) at an MOI of 0.08, approximately 72 h after RCAS(J)GFP infection (Fig. 1*A*). Based on this result, ChGeCKO cells were infected with RCAS(J)GFP at an MOI of 0.08. After 72 h of infection, EGFP-negative cells were collected using a flow cell sorter and challenged with RCAS(J)GFP. To improve the accuracy of screening, this process was repeated for three rounds. After the third round, the genomic DNA of EGFP-negative cells was extracted and sequenced to identify enriched genes that could potentially encode host factors essential for ALV-J infection. The number of significantly enriched genes in the first, second, and third rounds of screening was 222, 218, and 207, respectively (Fig. 1*B*). Notably, 42 identical candidates were significantly enriched after all three rounds of RCAS(J)GFP challenge (Fig. 1*B*).

To explore the biological functions of these candidate host genes, we performed gene ontology (GO) and Kyoto Encyclopedia of Genes and Genomes (KEGG) pathway enrichment analyses on the 42 identified genes. GO analysis demonstrated associations with several important biological processes, such as positive regulation of oxidative phosphorylation, regulation of multivesicular body size, positive regulation of the p38MAPK cascade, soft palate development, and rhombomere six development. Furthermore, cGMP-dependent protein kinase activity, RNA polymerase II CTD heptapeptide repeats, and cyclin-dependent protein serine/threonine kinase activity were enriched under the “molecular function” category, whereas lamellipodium, late endosome membrane, and ESCRT II complex were enriched under the “cellular component” category (Fig. 1*C*). KEGG analysis revealed that the majority of the target proteins were associated with the cell cycle, p53 signaling pathway, MAPK signaling pathway, cell migration, and oxidative phosphorylation (Fig. 1*D*). These findings suggest that the candidate host genes play a role in host cell cycle regulation, retroviral release, transcription, and DNA replication, all processes potentially important for ALV-J infection.

To validate the role of cell cycle regulation in ALV-J infection, we focused on three candidate genes: dihydrofolate reductase (*DHFR*), cyclin-dependent kinase 1 (*CDK1*), and *Cables1*. We constructed monoclonal gene knockout cell lines using the CRISPR/Cas9 system for these genes in DF-1 cells. Sequence analysis showed that the deletion of 14, 11, and 26 bases in the sequences of *Cables1*, *CDK1*, and *DHFR*, respectively, resulted in frameshift mutations (Fig. 1*E*), indicating that the three knockout cell lines were successfully constructed. Subsequently, we infected these knockout cell lines and WT DF-1 cells with RCAS(J)GFP to assess their differences in susceptibility to infection. Fluorescence intensity measurements at three days post-infection (dpi) showed that the knockout of *DHFR*, *CDK1*, and *Cables1* inhibited RCAS(J)GFP infection (Fig. 1*F*). Similarly, flow cytometry results demonstrated that at three dpi with an MOI of 0.01, the infection rates of RCAS(J)GFP were 22%, 23%, 20%, and 35% in *DHFR*, *CDK1*, and *Cables1* knockout, and WT DF-1 cells, respectively (Fig. 1*G*). Notably, a reduction of 40%, 36%, and 44% in RCAS(J)GFP infection rates was observed in *DHFR*, *CDK1*, and *Cables1* knockout lines, respectively, compared to that in WT DF-1 cells (Fig. 1*H*).

Taken together, these results indicate that the host factors DHFR, CDK1, and Cables1 are required for ALV-J replication and that cell cycle regulation-related genes are involved in ALV-J infection. Given the significant reduction in RCAS(J)GFP infection observed in *Cables1* knockout cells, we selected Cables1 for further investigation.

### Cables1 is a critical host factor for ALV-J replication

**Figure 2:**
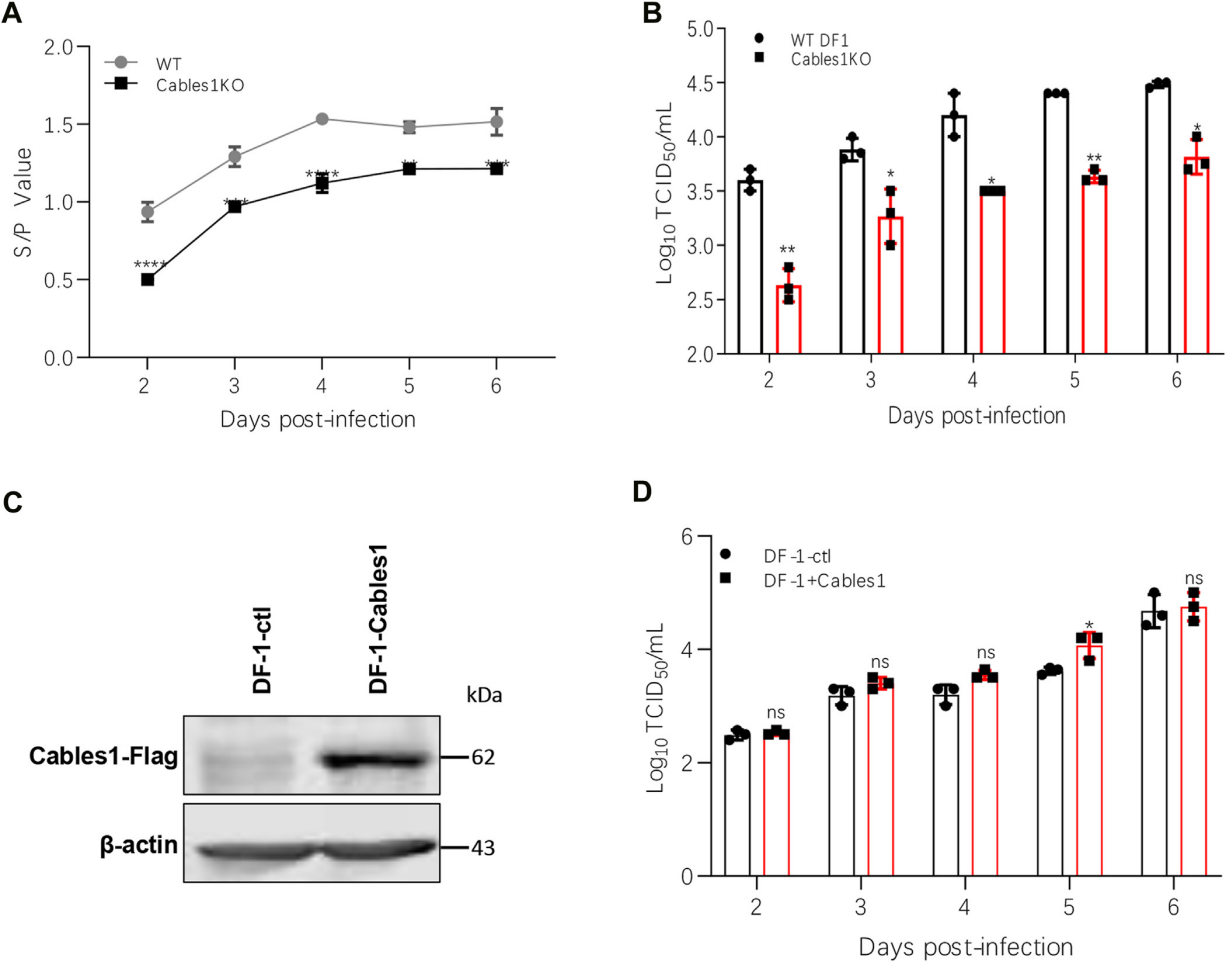
**Cables1 is a host factor required for ALV-J replication.***A*-*B*, knockout of Cables1 inhibits ALV-J HPRS103 replication. The WT DF-1 cells and *Cables1* knockout cell lines were infected with ALV-J HPRS103 strain at MOI of 0.01. The cells were harvested from 2 days to 6 days. The viral p27 expression level was determined based on the value of the ratio of the sample to the positive control (the standard p27 protein sample) (S/P) for p27 antigen detection (*A*), and viral titers were quantified by TCID_50_ assay (*B*). *C*-*D*, overexpression of Cables1 promoted replication of ALV-J HPRS103 strain. *C*, Western blotting assay to detect the Cables1-Flag protein. The Cables1-Flag overexpression cell lines were constructed by lentiviral expression systems, and the expression of Cables1-Flag was detected by Western blotting using mouse anti-Flag mAb. The WT DF-1 cells and *Cables1* overexpression cell lines were infected with ALV-J HPRS103 strain at an MOI of 0.01. The viral titers were quantified by TCID_50_ assay *D*, a representative experiment out of three repeats is shown, and data are shown as means ± SD for triplicates from the representative experiment. ∗, *p* < 0.05; ∗∗, *p* < 0.01; ∗∗∗, *p* < 0.001; ns, not significant.

To further verify the importance of Cables1 in ALV-J infection, we compared viral replication in WT DF-1 and *Cables1* knockout cells. Both cell lines were infected with the ALV-J prototype strain, HPRS103. Samples were collected daily from two to six dpi and analyzed for viral p27 expression by ELISA and viral titers by the median tissue culture infectious dose (TCID_50_) method. Compared with the control WT DF-1 cells, *Cables1* knockout cells displayed a reduction in viral p27 expression, ranging from 18% to 46%, from two to six dpi (Fig. 2*A*). Furthermore, TCID_50_ assays revealed that *Cables1* knockout mediated a 1.4-to 8.9-fold decrease in HPRS103 titers between two and six dpi (Fig. 2*B*), with the greatest reduction observed at two dpi. These results collectively demonstrate that the *Cables1* knockout significantly inhibited HPRS103 replication.

To further confirm the influence of Cables1 in promoting ALV-J infection, we generated DF-1 cell lines with Cables1 overexpression using a lentiviral expression system. Western blotting confirmed successful Cables1 overexpression (Fig. 2*C*). These Cables1-overexpressing and WT DF-1 cells were infected with HPRS103. Samples were collected daily from two to six dpi and analyzed by viral titers using TCID_50_. TCID_50_ assays indicated a 1.1-to 2.63-fold increase in viral titer compared with the control group (Fig. 2*D*). These results identify Cables1 as a potential host factor involved in ALV-J replication.

### Cables1 interacts with ALV-J p15

**Figure 3:**
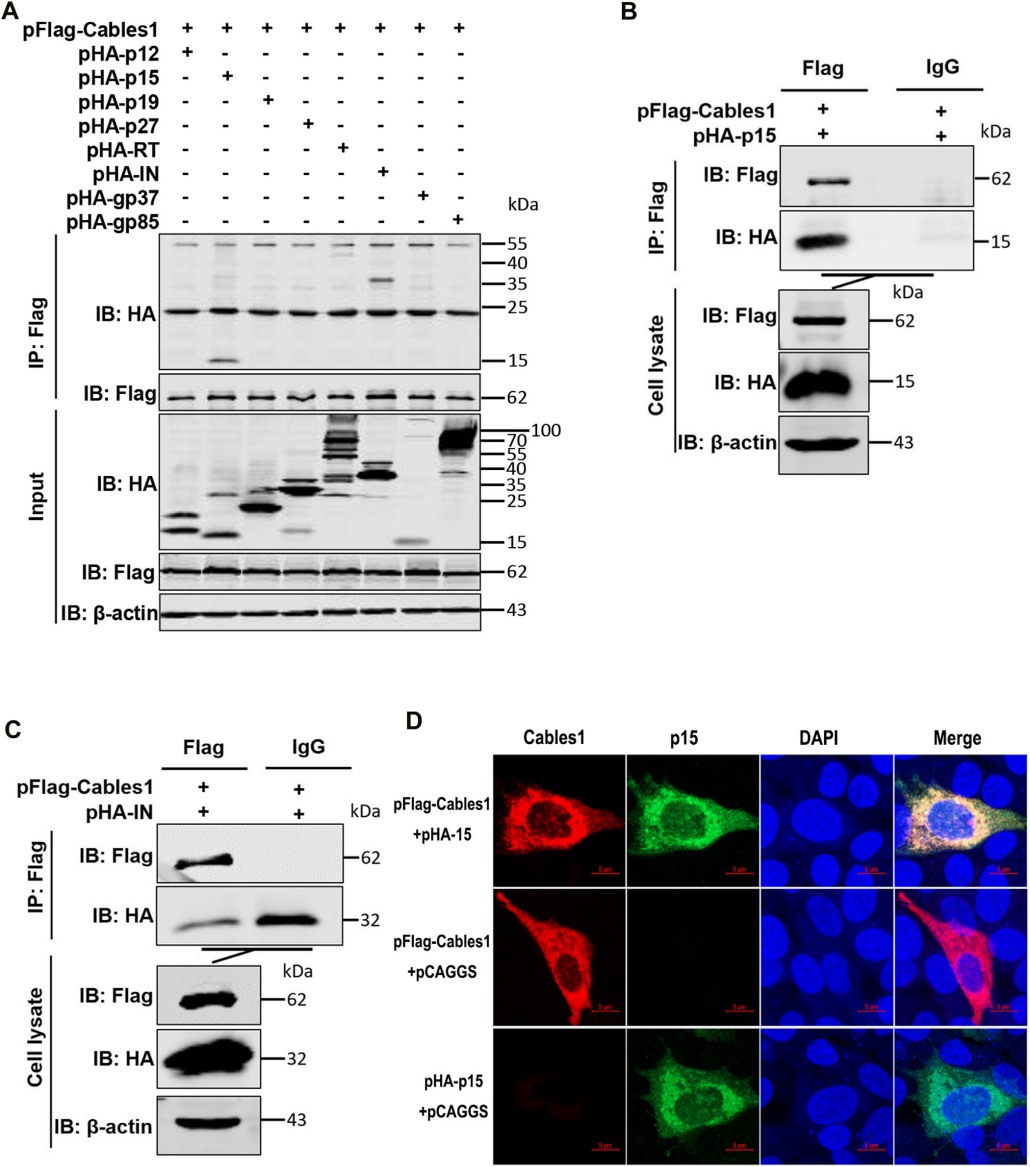
**Cables1 interacted with ALV-J p15.***A*-*C*, interaction between Cables1 and viral proteins was detected by Co-IP in HEK293T cells. *A*, HEK293T cells were co-transfected with pFlag-Cables1 and pHA-p12, pHA-p15, pHA-p19, pHA-p27, pHA-IN, pHA-RT, pHA-gp37, pHA-gp85, respectively. After 36 h, the cells were harvested and lysed. The lysates were incubated with anti-Flag mAb and detected with the indicated antibodies. *B* and *C*, HEK293T cells were co-transfected with pFlag-Cables1 and pHA-p15 or pHA-IN for 36 h. The lysates were incubated with anti-Flag mAb or mouse-derived IgG. *D*, confocal assays were used to assess the co-localization between Cables1 and p15. DF-1 cells were co-transfected with pFlag-Cables1 and pHA-p15. After 24 h following transfection, the cells were harvested and incubated with the corresponding antibodies. The interaction between Cables1 and p15 was analyzed *via* confocal assay. Scale bar: 5 μm. A representative experiment out of three repeats is shown.

To test whether Cables1-mediated ALV-J replication is facilitated *via* direct interaction with viral proteins, we co-transfected human embryonic kidney 293T (HEK293T) cells with a plasmid expressing Flag-tagged Cables1 (pFlag-Calses1) and HA-tagged versions of various ALV-J proteins (pHA-p19, pHA-12, pHA-p27, pHA-p15, pHA-RT, pHA-IN, pHA-gp37, and pHA-gp85) for 36 h. We detected the protein interactions using a co-immunoprecipitation (Co-IP) assay. The results showed that Cables1 specifically interacted with p15 and IN but not with other viral proteins (Fig. 3*A*). Further verification of these interactions was done using a mouse anti-Flag monoclonal antibody (mAb) and mouse control IgG. Western blotting analysis revealed a specific interaction between Cables1 and p15 (Fig. 3*B*). Unexpectedly, IN appeared to interact with the control IgG, indicating nonspecific binding between IN and the mouse anti-Flag mAb (Fig. 3*C*). Furthermore, confocal microscopy results confirmed that Cables1 colocalized with p15 in the cytoplasm (Fig. 3*D*). These results indicated that Cables1 interacted with ALV-J p15, which was selected for further study.

### Cables1 promotes K26-linked ubiquitination of ALV-J p15

**Figure 4:**
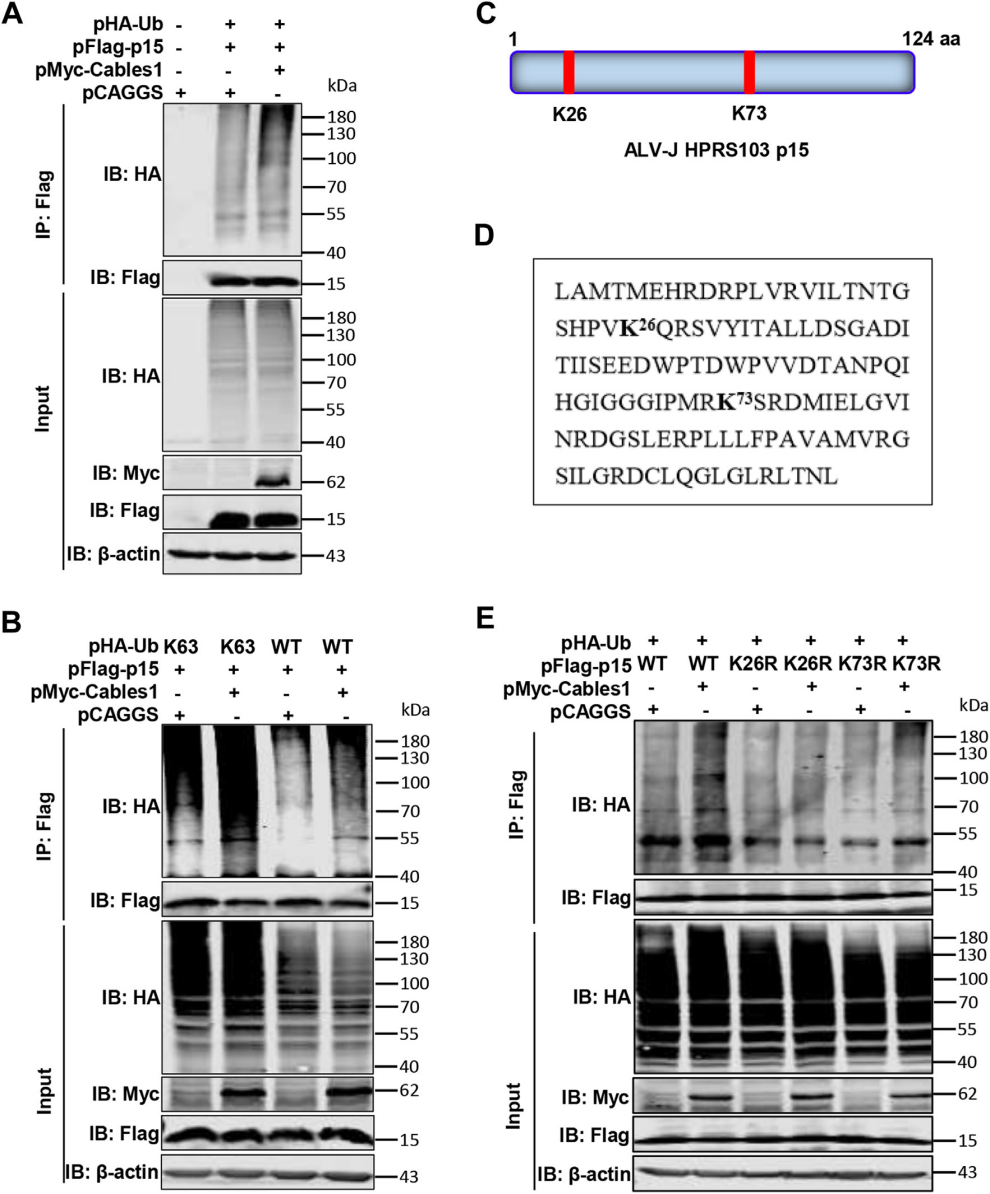
**Cables1 enhanced ALV-J p15 ubiquitination *via* Lys26.***A*, cables1 promoted ALV-J p15 ubiquitination. The pHA-Ub and pFlag-p15 plasmids were co-transfected with or without pMyc-Cables1 into HEK293T cells for 36 h. Empty vector pCAGGS (eukaryotic expression-empty vector) was transfected into HEK293T cells for 36 h as negative control. The lysates were incubated with anti-Flag mAb and detected using the corresponding antibody. *B*, cables1 enhanced the K63-linked polyubiquitination of ALV-J p15. pFlag-p15 and pHA-Ub-K63 (the ubiquitin with all lysine substituted by arginine but for the lysine 63 site) plasmids were co-transfected with pMyc-Cables1 or empty vector pCAGGS into HEK293T cells for 36 h. Then, the lysates were incubated with indicated mouse anti-Flag mAb and detected using IP. *C* and *D*, schematic representation of the potential ubiquitination site of ALV-J p15. And the p15 residual sequence from residues 1 to 124 of ALV-J p15. Lysine residues are displayed in boldface. *E*, Lys26 is the key site for the increase in ALV-J p15 ubiquitination mediated by Cables1. The ALV-J p15 plasmids including the WT and different mutants and pHA-Ub were co-transfected with pMyc-Cables1 or empty vector pCAGGS into HEK293T cells for 36 h. Then, the lysates were detected by immunoblotting. A representative experiment out of three repeats is shown.

Cables1 functions as an adaptor protein, known to mediate protein modifications through interacting with target proteins (23). Notably, ALV-J Gag can be ubiquitinated, with p15 potentially serving as the target substrate at the C-terminus of Gag (24). To elucidate whether Cables1 could promote the ubiquitination of p15, we transfected HEK293T cells with plasmids expressing Flag-tagged p15 (pFlag-p15) and either HA-tagged ubiquitin (pHA-Ub) alone or together with Myc-tagged Cables1 (pMyc-Cables1). Western blotting results indicated that p15 could be ubiquitinated, and importantly, overexpression of Cables1 promoted the ubiquitination (Fig. 4*A*). K63-linked ubiquitination of target proteins typically mediates protein function. To determine if p15 undergoes K63-linked ubiquitination, we transfected HEK293T cells with pFlag-p15, pHA-Ub-K63 (the ubiquitin with all lysine substituted by arginine but for the lysine 63 site), and pMyc-Cables1. Furthermore, Co-IP and ubiquitination assay results showed that p15 was modified by K63-linked ubiquitin, and this ubiquitination increases upon overexpression of Cables1 (Fig. 4*B*). As shown in Fig. 4, *C* and *D*, there are two potential ubiquitination sites (K26 and K73) within p15. To identify the specific site targeted by Cables1, we generated p15 mutants where K26 and K73 were substituted with arginine and co-transfected with pMyc-Cables1 and pHA-Ub in HEK293T cells. As shown in Fig. 4*E*, in the context of Cables1 overexpression, substitution of K26 significantly reduced the Cables1-mediated increase in p15 ubiquitination based on the comparison analysis between the fourth lane and third lane (Fig. 4*E*). However, substitution of K73 did not affect p15 ubiquitination promoted by Cables1 under Cables1 overexpression according to the comparison analysis between the sixth lane and fifth lane (Fig. 4*E*). These results suggest that ALV-J p15 undergoes ubiquitination and that Cables1 enhances this process by targeting the K26 site.

### Mutation at the ALV-J p15 K26 site attenuates replication of ALV-J

**Figure 5:**
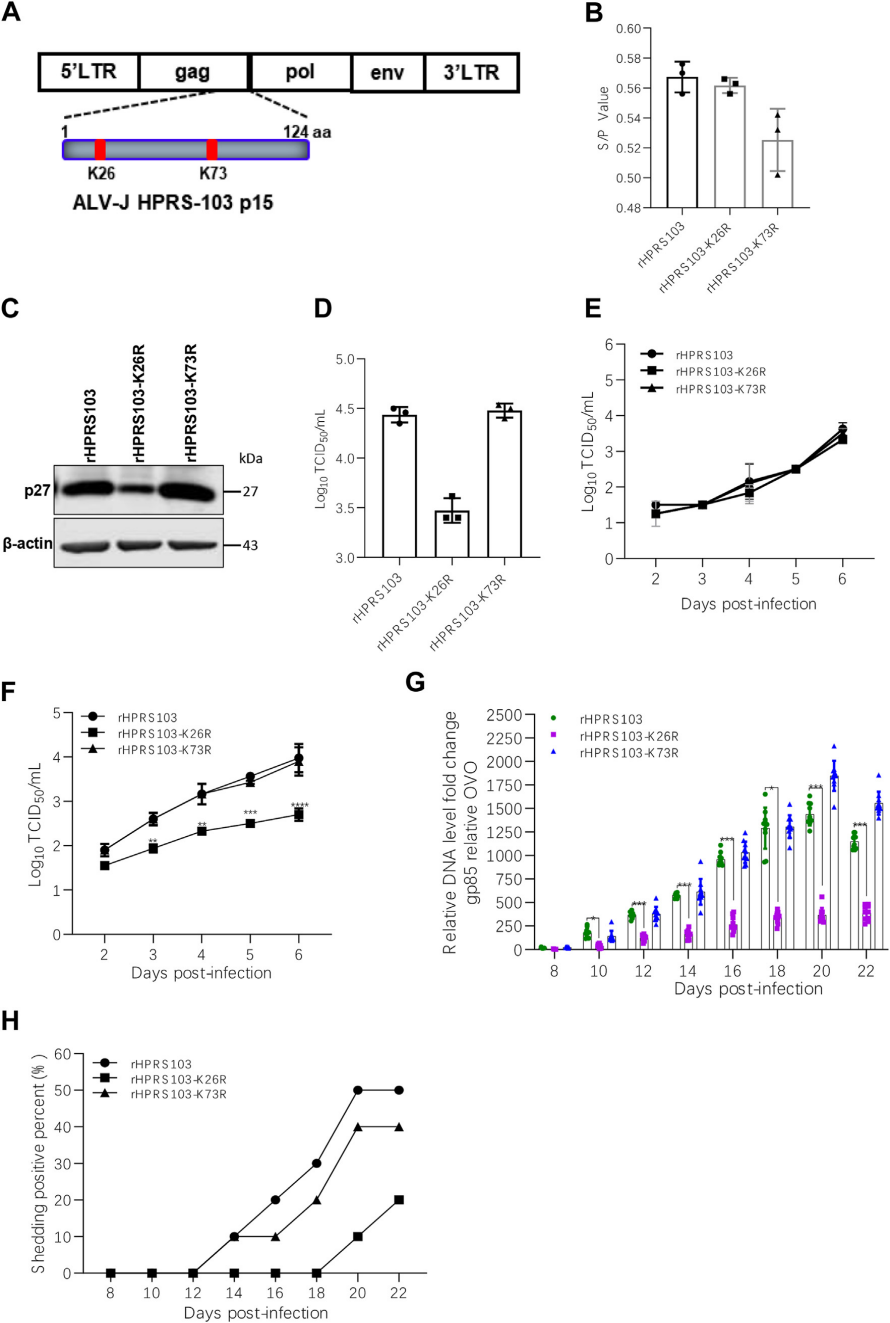
**The replication ability of recombinant viruses rHPRS103-K26R, rHPRS103-K73R, and rHPRS103 under *in vivo* and *in vitro* conditions.***A*, the schematic diagram shows the construction of the recombinant viruses rHPRS103-K26R and rHPRS103-K73R. Lys26 and Lys73 of rHPRS103 p15 were mutated to Arg for constructing the recombinant virus rHPRS103-K26R and rHPRS103-K73R, respectively, based on the rHPRS103 backbone. *B*, ELISA assay. The rescued viruses rHPRS103-K26R, rHPRS103-K73R, and rHPRS103 were detected with mouse anti-p27 mAb, rHPRS103 as a reference. *C*, Western blotting. DF-1 cells were infected with recombinant viruses rHPRS103-K26R, rHPRS103-K73R, and rHPRS103 and then analyzed with an anti-p27 monoclonal antibody at 72 h following infection. *D*-*F*, TCID_50_ assay. The rescued viruses rHPRS103-K26R, rHPRS103-K73R, and rHPRS103 were titrated using the Reed-Muench method (*D*). The replication activities of rHPRS103-K26R, rHPRS103-K73R, and rHPRS103 were detected using the TCID_50_ assay *in vitro*. *Cables1* knockout cells (*E*) and WT DF-1 cells (*F*) were infected with rHPRS103-K26R, rHPRS103-K73R, and rHPRS103 at MOI of 0.01. Subsequently, the samples were harvested at two, three, four, five and six dpi and titrated using the Reed-Muench method. *G*, One-day-old SPF chicks were injected intraabdominally with rHPRS103-K26R, rHPRS103-K73R, and rHPRS103 (n = 10, dose=5000 TCID_50_). The whole-blood samples collected from SPF chicks infected with rHPRS103-K26R, rHPRS103-K73R, and rHPRS103 were examined for viral loads using real-time SYBR qPCR mix PCR at different time points. OVO: chicken ovotransferrin gene. *H*, cloacal swabs collected from SPF chicks infected with the rHPRS103-K26R, rHPRS103-K73R, and rHPRS103 were evaluated by ELISA at different time points. A representative experiment out of three repeats is shown, and data are shown as means ± SD for triplicates from the representative experiment. ∗, *p* < 0.05; ∗∗, *p* < 0.01; ∗∗∗, *p* < 0.001; ns, not significant.

To further investigate the importance of the K26 and K73 ubiquitination sites on p15 in HPRS103 replication, two ALV-J variants (rHPRS103-K26R and rHPRS103-K73R) comprising K26R or K73R mutations in p15 were constructed and rescued using rHPRS103 as the backbone (Fig. 5*A*). ELISA results showed that the rescued viruses had a higher expression of p27 (Fig. 5*B*). Similarly, western blotting revealed strong p27 expression after rHPRS103-K26R and rHPRS103-K73R infection in DF-1 cells (Fig. 5*C*). Furthermore, TCID_50_ assay showed that the virus titers of rHPRS103-K26R, rHPRS103-K73R, and rHPRS103 were 10^3.5^, 10^4.5^ and 10^4.4^ TCID_50_/ml, respectively (Fig. 5*D*), indicating all viruses were successfully rescued.

To further identify the role of Cables1 in ALV-J infection, *Cables1* knockout DF-1 cells were infected with rHPRS103, rHPRS103-K26R, and rHPRS103-K73R at an MOI of 0.01 and harvested to detect viral titer using TCID_50_ assay from two to six dpi. The results showed the replication capacity among the three viruses is no significant difference in *Cables1* knockout DF-1 cells (Fig. 5*E*), indicating that Cables1 is key host factor for viral replication.

To further compare the replication ability of the rescued rHPRS103-K26R, rHPRS103-K73R, and rHPRS103, DF-1 cells were infected with each virus at an MOI of 0.01, and viral titers were measured daily from two to six dpi. The replication kinetics curve showed no significant difference in viral replication between rHPRS103-K73R and rHPRS103. However, the viral titers of rHPRS103-K26R were lower (2.2-to 18.8-fold decrease) compared to both rHPRS103 and rHPRS103-K73R across all time points (Fig. 5*F*). These findings indicate that ubiquitination of ALV-J p15 at the K26 site is important for efficient viral replication.

To further examine the influence of the p15 K26 site on the replication ability of ALV-J *in vivo*, we infected one-day-old specific pathogen-free (SPF) chicks with the three viruses *via* intraperitoneal injection at a dose of 5000 TCID_50_. Viral shedding and viremia were detected by collecting cloacal swabs and uncoagulated blood samples, respectively, at 8, 10, 12, 14, 18, 20, and 22 dpi. Viral loads of blood in rHPRS103-K26R group were 2.7- to 5-fold lower than that of rHPRS103 group from 10 to 22 dpi (Fig. 5*G*). Moreover, viral shedding in chicks infected with rHPRS103-K26R was detected later (18 dpi) compared to those infected with rHPRS103 and rHPRS103-K73R (12 dpi) (Fig. 5*H*). Additionally, the proportion of cloacal swabs that tested positive for ALV was lower in the rHPRS103-K26R group (20%) compared to the rHPRS103 and rHPRS103-K73R groups (50%) (Fig. 5*H*). Taken together, these results indicate that the K26 ubiquitination of p15 is important for ALV-J replication both *in vivo* and *in vitro*.

## Discussion

ALV-J is a retrovirus characterized by its high dependence on host factors for various aspects of its infection cycle. Studying the interaction between ALV-J and host factors is crucial not only for understanding the viral replication mechanism within host cells but also for guiding the development of novel strategies for ALV-J prevention and control as well as elucidating specific antiviral mechanisms. In this study, we used the ChGeCKO library to identify 42 candidate host factors potentially associated with ALV-J replication. Subsequently, we narrowed down on Cables1 as an important host gene involved in ALV-J replication. Furthermore, we found that Cables1 promoted ALV-J replication by directly interacting with ALV-J p15 to enhance its ubiquitination, a process essential for viral replication in both *in vivo* and *in vitro* models.

CRISPR screening offers a valuable method for the identification of host genes associated with viral infections. This method has high concordance in comparison to siRNA screening (25, 26), which has been widely used in mammalian cells, and has demonstrated remarkable progress in uncovering novel host factors required for viral replication (16, 17, 18, 19, 20, 21). Furthermore, CRISPR knockout screening method may be more effective than siRNA screening method due to the potential for a long half-life of the target protein or transcript (27).

It is important to note that while the whole chicken genome was sequenced years ago (28), there are few reports that have examined whole chicken genome libraries based on CRISPR screening (29). Since ALV-J-infected DF-1 cells do not cause a cytopathic effect (CPE), we implemented ChGeKO screening using an ALV-J reporter virus and relied on EGFP-negative cells as the selection criterion. To minimize false positives, we performed three rounds of screening. Subsequently, 42 identical host genes were enriched, indicating that our screening method was reliable and reproducible and serves as a reference for screening host factors for avian pathogens without CPE. Importantly, the results of our screen provide the groundwork for further investigation into the molecular mechanisms underlying ALV-J replication.

ALV-J infection promotes cell proliferation and cell cycle progression through indirect regulation of cell cycle regulation-related genes by microRNAs, such as micoRNA-221, in DF-1 cells (30). Moreover, ALV-J downregulates the expression of the chicken 14-3-3σ protein (known to downregulate the expression of CDK2/CDC2), thereby promoting cell proliferation (31). Cell cycle regulation-related genes, such as those involved in mediating HIV infection, are required for retroviral replication (22). In this study, our screening revealed that cell cycle regulators, including *CDK1*, *DHFR*, and *Cables1*, are required for ALV-J replication. Similarly, *CDK7*, *CDK9*, and *CDK11* are involved in the transcription and processing of viral RNA during the early stages of HIV-1 replication (32, 33, 34). These results further emphasize the critical role of cell cycle regulation genes in retroviral replication.

Cables1 was first identified as an adaptor protein that bridges the interaction between Cdk5 and Abl, promoting Cdk5 phosphorylation in neurons (23). It is a multifunctional protein involved in the induction of cell death by functionally connecting p53 or p73 (35), influencing the cell cycle by enhancing CDK2 phosphorylation (36), and stabilizing p21 by antagonizing proteasome subunit alpha type three (37). Our findings elucidate a novel role for chicken Cables1 in promoting ALV-J replication. As an adaptor protein, Cables1 directly interacts with the ALV-J p15 protein, leading to increased p15 ubiquitination. To our knowledge, this is the first report demonstrating the requirement of Cables1 in ALV-J replication in chicken.

The ubiquitination of viral proteins plays an important biological role in viral replication. Infectious bursal disease virus (IBDV) VP1 is modified by polyubiquitination, which is important for facilitating VP1’s polymerase activity (38). The avian influenza A virus NP protein is modified by mono-ubiquitination, which is crucial for viral RNA replication (39). Moreover, the ubiquitination of the retroviral Gag protein is vital for virion budding from the host cells. Ubiquitination of HIV Gag enhances the binding between Tsg101 and the late domain of Gag to promote budding and release (40, 41). Budding of Rous sarcoma virus (RSV), a member of avian retroviruses, requires cellular Nedd4-family E3 ubiquitin ligases to ubiquitinate the p2b region of Gag (42). Inhibition of RSV budding by proteasome inhibitors results in the accumulation of Gag on the plasma membrane; however, overexpression of ubiquitin restores RSV budding (43). A previous study indicated that ALV-J Gag can be ubiquitinated and that p15 may serve as a potential target substrate (24). Our findings confirm that ALV-J p15 undergoes polyubiquitination, and this process is enhanced by overexpression of Cables1. Furthermore, our *in vivo* and *in vitro* results indicate that ubiquitination of ALV-J p15 is important for efficient ALV-J replication. However, whether the ubiquitination of ALV-J p15 is vital for viral budding requires further examination.

In summary, we performed ChGeCKO screening in DF-1 cells for the identification of host factors associated with ALV-J infection and identified Cables1 as a critical host factor required for ALV-J replication in chickens. In addition, our findings indicate that Cables1 promotes ALV-J replication by enhancing the ubiquitination of p15 *via* the Lys26 site. Therefore, Cables1 holds promise as a potential target for gene editing to combat ALV-J infection in chickens.

## Experimental procedures

### Cells and viruses

HEK293T and DF-1 cells were cultured in Dulbecco’s Modified Eagle’s Medium (DMEM), supplemented with 10% fetal bovine serum (FDN500, Excell Bio) and 100 mg/ml of penicillin and streptomycin, and incubated at 37 °C with 5% CO_2_. The ALV-J prototype strain, HPRS103, was kindly provided by Venugopal Nair (Pirbright Institute) and propagated in DF-1 cells. RCAS(J)GFP recombinant virus, ALV-J reporter virus, was constructed as previously described (44) and stored by our laboratory (the construction information has been described in construction of plasmids section).

### Construction of plasmids

Cables1 was amplified from DF-1 cDNA by PCR and inserted into a pCAGGS plasmid with a Flag tag at the C-terminus. In order to detect the interaction between Cables1 and the viral proteins of HPRS103, p12, p15, p19, p27, RT, and IN, gp85 and gp37, without their intracellular region and transmembrane domain, were amplified from proviral DNA and inserted into a pCAGGS plasmid with an HA tag at the C-terminus. To determine the ubiquitination of p15, pMyc-Cables1 and pFlag-p15 were constructed by fusing Flag or Myc tags at their C-termini. pHA-Ub was purchased from GenScript (Shanghai). The K63 mutant plasmid (pHA-UbK63) was constructed by replacing the all lysine of pHA-Ub with arginine but for 63rd lysine residue. To investigate the ubiquitination of p15 by Cables1, two Flag-tagged p15 mutant plasmids (pFlag-p15K26R and pFlag-VP3K73R) were constructed by substituting the 26th and 73rd lysine residues with arginine in pFlag-p15.

RCAS(J)GFP was constructed as previously described (44). In brief, the pRCAS(A)-GFP vector, gifted by Stephen Hughes professor in the National Institutes of Health, was digested by *Kpn* I and *Stu* I to remove the *env* gene of Ross sarcoma virus A. Subsequently, the *env* gene of ALV-J HPRS103 was amplified by primers and digested by *Kpn* I and *Stu* I. Finally, the *env* gene of ALV-J HPRS103 was introduced into the pRCAS(A)-GFP vector by T4 ligase, and named pRCAS(J)-GFP vector.

### Generation of DHFR, CDK1, and Cables1 knockout DF-1 cell lines

The *DHFR*, *CDK1*, and *Cables1* knockout cell lines were generated using CRISPR/Cas9 gene editing. Briefly, guide RNA (gRNA) target sites for the genes were designed using E-CRISPR (http://www.e-crisp.org/E-CRISP/designcrispr.html). Subsequently, the amplified DNA fragments, including the U6 promoter, target RNA sites, and gRNA scaffold, were introduced into the pMD18-T vector (TAKARA, Japan) and named pMD18-T-gRNA. DF-1 cells were co-transfected with pMD18-T-gRNA and pMJ920 (Addgene: #42234) using the TransIT-X2 Kit (MIR 6000; Mirus Bio LLC, USA), as per the manufacturer’s instructions. After 48 h, EGFP-positive cells were sorted into 96-well plates by a flow cytometry-based cell sorter. Single-cell clones were screened, identified by sequence analysis, and expanded in culture. All knockout cell lines were culture-negative for *Mycoplasma*.

### Generation of Cables1-overexpressing cells

pLVX-IRES-zsGreen-Cables1-Flag was co-transfected with psPAX2 (a gift from Didier Trono; Addgene plasmid # 12260; http://n2t.net/addgene:12260; RRID: Addgene_12260) and pVSV-G (a gift from Akitsu Hotta; Addgene plasmid # 138479; http://n2t.net/addgene:138479; RRID: Addgene_138479) into HEK293T cells using a polyethyleneimine transfection reagent (Polysciences), following the manufacturer's instructions. After 48 h, the culture supernatants were collected, clarified by low-speed centrifugation (300*g*), and incubated with DF-1 cells in six-well plates. After 24 h, cells showing red fluorescence were sorted into 96-well plates by a flow cytometry-based cell sorter. Single-cell clones were screened, and the overexpression was confirmed by western blotting using corresponding antibodies. The Cables1-overexpressing cells were culture negative for *Mycoplasma*.

### Co-IP assay

HEK293T cells were cultured in a six-well plate and transfected with the corresponding plasmids. After 36 h of transfection, the cells were washed thrice with PBS and lysed in lysis buffer (P0013F; Beyotime) for 30 min. The supernatants from these cell lysates were incubated with anti-Flag mouse mAb (F1804, SLCD6338, Sigma-Aldrich) or control mouse IgG for 6 h at 4 °C. Subsequently, 25 μl of protein A/G agarose (A10001, Abmart) was added to the lysate mixture, followed by incubation for 6 h. The samples were then centrifuged at 4 °C and washed five times with ice-cold PBS. The samples were boiled at 100 °C for 15 min and separated by sodium dodecyl sulfate-polyacrylamide gel electrophoresis (SDS-PAGE) to detect target proteins by western blotting.

### Confocal microscopy

A monolayer of DF-1 cells was cultured in 12-well plates and co-transfected with pFlag-Cables1 and pHA-p15 using the TransIT-X2 kit, as per the manufacturer’s instructions. After 24 h, the cells were washed thrice with PBS and then fixed using 4% (v/v) paraformaldehyde for 1 h at 37 °C. After washing thrice with PBS, the cells were permeabilized using 0.2% Triton X-100 for 15 min at 37 °C. After washing thrice with PBS again, the cells were blocked with 5% (w/v) skim milk for 1.5 h at 37 °C. Subsequently, the cells were washed thrice with PBS and incubated with mouse anti-HA mAb (H9658, Sigma-Aldrich) and rabbit anti-Flag mAb (F2555, Sigma-Aldrich) for 2 h at 37 °C. After washing thrice with PBS, the cells were incubated with Alexa Fluor 546 goat anti-rabbit IgG (A11035, Invitrogen) and Alexa Fluor 488 goat anti-mouse IgG (A11029, Invitrogen) for 1 h. The cells were stained with DAPI (C1005, Beyotime) for 15 min at 37 °C, and examined under a laser confocal microscope (LSM980; Zeiss).

### Western blotting

The protein samples boiled with 5 × SDS loading buffer (P0015L, Beyotime) were separated by 10% SDS-PAGE gels and transferred onto a nitrocellulose membrane (Hybond-C Super; GE Healthcare). The membrane was blocked with 5% (w/v) skimmed milk for 2 h and incubated with the monoclonal or polyclonal antibodies for 1.5 h. Subsequently, it was washed three times with PBST. Then, the membranes were incubated with IRDye 800CW goat anti-mouse IgG (H  + L) antibody for 1 h. Finally, the membrane blots were scanned using an Odyssey infrared imaging system (LI-COR Biosciences).

### TCID_50_ titration

DF-1 cells were cultured in a 96-well plate to determine the titers of ALV-J HPRS103 and its mutants. The supernatants of infected cells were harvested at different time points and incubated with DF-1 cells in a series of ten-fold dilutions. The titers of the supernatants (TCID_50_) were calculated using the Reed–Muench method.

### Rescue of recombinant viruses

The pRCAS(J)-GFP, pBlue-HPRS103 plasmid (the infectious clone of ALV-J HPRS103) and its mutant plasmids, pBlue-HPRS103-K26R and pBlue-HPRS103-K73R, were transfected into DF-1 cells using the TransIT-X2 kit, as per the manufacturer’s instructions. After 7 days, the culture supernatants were harvested and subjected to blind passaging for four generations to obtain secondary DF-1 cells. The rescued viruses were identified by ELISA and western blotting, and their titers (TCID_50_) were determined using the Reed–Muench method. The rescued viruses were named RCAS(J)GFP, rHPR103, rHPRS103-K26R, and rHPRS103-K73R.

### ALV-J infection

WT DF-1 cells, *Cables1* knockout cells, *CDK1* knockout cells, and *DHFR* knockout cells were seeded into the plates. The cells were infected with RCAS(J)GFP when they were at more than 90% confluence. After 72 h, the infected cells were collected to analyze GFP fluorescence from the virus by flow cytometry. Furthermore, DF-1 cells and *Cables1* knockout cells were infected with ALV-J HPRS103 at an MOI of 0.01 to identify the regulatory role of Cables1 in ALV-J replication. Moreover, *Cables1* over-expression cells were infected with rHPRS103, rHPRS103-K26R, and rHPRS103-K73R at an MOI of 0.01 to identify the role of Cables1 in ALV-J infection.

### Animal experiments

To examine the replication characteristics of rHPRS103-K26R, rHPRS103-K73R, and rHPRS103 *in vivo*, thirty 1-day-old SPF chicks were randomly divided into three groups and infected with rHPRS103-K26R, rHPRS103-K73R, and rHPRS103 at 5000 TCID_50_. After viral infection, cloacal swabs and anticoagulated blood from SPF chicks infected with the viruses were collected at 8, 10, 12, 14, 18, 20, and 22 dpi, respectively. Viral DNA extracted from anticoagulant-treated blood was detected *via* real-time PCR (qPCR). The virus shedding level was assessed using an Avian Leukosis Virus Antigen Test (ELISA) kit (NEE83500, Sinaean Biologics) to examine the cloacal swabs according to the manufacturer’s instructions. All animal experiments were approved by the Committee on the Ethics of Animal Experiments at the Harbin Veterinary Research Institute, Chinese Academy of Agricultural Sciences and were performed according to the guidelines for experimental animals of the Ministry of Science and Technology.

### qPCR

To detect viral loads in whole-blood samples from 8 to 22 dpi, DNA was extracted using a TIANamp Blood DNA Kit (TIANGEN, China). Viral loads in whole-blood samples were detected by real-time PCR using the THUNDERBIRD SYBR q-PCR Mix Kit (QPS-201, TOYOBO). qPCR was used to detect the expression of ALV-J gp85 and the chicken ovotransferrin gene (OVO, housekeeping genes) in the sample (45, 46). qPCR was performed using a fluorescent qPCR instrument (Quant Studio 5, Applied Biosystems Waltham). The relative expression levels of gp85 were calculated with the comparative *C*_*T*_ (2^−ΔΔCt^) method. The real-time PCR conditions and protocols have been previously described (47).

### Statistical analysis

All assays were performed independently at least three times. Prism software (Version 8.0) was employed for statistical analysis, and the Student’s t-tests were performed to establish differences between groups. Statistical significance was set at *p* < 0.05.

## Data availability

All data generated are contained in the article.

## Conflict of interest

The authors declare that they have no conflicts of interest with the contents of this article.

## References

[1] Payne L.N., Nair V. (2012). The long view: 40 years of avian leukosis research. Avian Pathol..

[2] Payne L.N., Howes K., Gillespie A.M., Smith L.M. (1992). Host range of Rous sarcoma virus pseudotype RSV(HPRS-103) in 12 avian species: support for a new avian retrovirus envelope subgroup, designated J. J. Gen. Virol..

[3] Li X., Yu Y., Ma M., Chang F., Muhammad F., Yu M. (2021). Molecular characteristic and pathogenicity analysis of a novel multiple recombinant ALV-K strain. Vet. Microbiol..

[4] Wang Q., Gao Y., Wang Y., Qin L., Qi X., Qu Y. (2012). A 205-nucleotide deletion in the 3' untranslated region of avian leukosis virus subgroup J, currently emergent in China, contributes to its pathogenicity. J. Virol..

[5] Sun H., Qin M., Xiao Y., Yang F., Ni W., Liu S. (2010). Haemangiomas, leiomyosarcoma and myeloma caused by subgroup J avian leukosis virus in a commercial layer flock. Acta Vet. Hung.

[6] Jiang L., Zeng X., Hua Y., Gao Q., Fan Z., Chai H. (2014). Genetic diversity and phylogenetic analysis of glycoprotein gp85 of avian leukosis virus subgroup J wild-bird isolates from Northeast China. Arch. Virol..

[7] Zeng X., Liu L., Hao R., Han C. (2014). Detection and molecular characterization of J subgroup avian leukosis virus in wild ducks in China. PLoS One.

[8] Gao Y., Yun B., Qin L., Pan W., Qu Y., Liu Z. (2012). Molecular epidemiology of avian leukosis virus subgroup J in layer flocks in China. J. Clin. Microbiol..

[9] Bai J., Howes K., Payne L.N., Skinner M.A. (1995). Sequence of host-range determinants in the env gene of a full-length, infectious proviral clone of exogenous avian leukosis virus HPRS-103 confirms that it represents a new subgroup (designated J). J. Gen. Virol..

[10] Vogt V.M. (1997). Retroviruses.

[11] Smith J.G., Cunningham J.M. (2007). Receptor-induced thiolate couples Env activation to retrovirus fusion and infection. PLoS Pathog..

[12] Barnard R.J., Young J.A. (2003). Alpharetrovirus envelope-receptor interactions. Curr. Top. Microbiol. Immunol..

[13] Tang S., Leng M., Tan C., Zhu L., Pang Y., Zhang X. (2023). Critical role for ribonucleoside-diphosphate reductase subunit M2 in ALV-J-induced activation of Wnt/β-catenin signaling via interaction with P27. J. Virol..

[14] Chai N., Bates P. (2006). Na+/H+ exchanger type 1 is a receptor for pathogenic subgroup J avian leukosis virus. Proc. Natl. Acad. Sci. U. S. A..

[15] Ramage H., Cherry S. (2015). Virus-host interactions: from unbiased genetic screens to function. Annu. Rev. Virol..

[16] Han J., Perez J.T., Chen C., Li Y., Benitez A., Kandasamy M. (2018). Genome-wide CRISPR/Cas9 screen identifies host factors essential for influenza virus replication. Cell Rep.

[17] Li B., Clohisey S.M., Chia B.S., Wang B., Cui A., Eisenhaure T. (2020). Genome-wide CRISPR screen identifies host dependency factors for influenza A virus infection. Nat. Commun..

[18] Wei J., Alfajaro M.M., DeWeirdt P.C., Hanna R.E., Lu-Culligan W.J., Cai W.L. (2021). Genome-wide CRISPR screens reveal host factors critical for SARS-CoV-2 infection. Cell.

[19] Wang R., Simoneau C.R., Kulsuptrakul J., Bouhaddou M., Travisano K.A., Hayashi J.M. (2021). Genetic screens identify host factors for SARS-CoV-2 and common cold coronaviruses. Cell.

[20] Bai J., Li K., Tang W., Liang Z., Wang X., Feng W. (2019). A high-throughput screen for genes essential for PRRSV infection using a piggyBac-based system. Virology.

[21] Jiang J., Sun Y., Wang Y., Sabek A., Shangguan A., Wang K. (2022). Genome-wide CRISPR/Cas9 screen identifies host factors important for porcine reproductive and respiratory syndrome virus replication. Virus Res..

[22] Shen H.H., Zhao Q., Wen Y.P., Wu R., Du S.Y., Huang X.B. (2023). Porcine reproductive and respiratory syndrome virus upregulates SMPDL3B to promote viral replication by modulating lipid metabolism. iScience.

[23] Zukerberg L.R., Patrick G.N., Nikolic M., Humbert S., Wu C.L., Lanier L.M. (2000). Cables links Cdk5 and c-Abl and facilitates Cdk5 tyrosine phosphorylation, kinase upregulation, and neurite outgrowth. Neuron.

[24] Wang Z., Hou X., Wang Y., Xu A., Cao W., Liao M. (2017). Ubiquitination of non-lysine residues in the retroviral integrase. Biochem. Biophysical Res. Commun..

[25] Srivastava K., Pandit B. (2023). Genome-wide CRISPR screens and their applications in infectious disease. Front. Genome editing.

[26] Bushman F.D., Malani N., Fernandes J., D'Orso I., Cagney G., Diamond T.L. (2009). Host cell factors in HIV replication: meta-analysis of genome-wide studies. PLoS Pathog..

[27] Flint M., Chatterjee P., Lin D.L., McMullan L.K., Shrivastava-Ranjan P., Bergeron É. (2019). A genome-wide CRISPR screen identifies N-acetylglucosamine-1-phosphate transferase as a potential antiviral target for Ebola virus. Nat. Commun..

[28] International Chicken Genome Sequencing Consortium (2004). Sequence and comparative analysis of the chicken genome provide unique perspectives on vertebrate evolution. Nature.

[29] Xu J., Liu Z., Liu Y., Liao Y., Qiu X., Song C. (2022). Development and preliminary application of chicken fibroblast cell line based on genome-scale Crispr-Cas9 Knockout screening. Chin. J. Anim. Infect. Dis..

[30] Ren C., Yu M., Zhang Y., Fan M., Chang F., Xing L. (2018). Avian leukosis virus subgroup J promotes cell proliferation and cell cycle progression through miR-221 by targeting CDKN1B. Virology.

[31] Wang M., Li H., Sun X., Qiu J., Jing C., Jia H. (2023). J subgroup avian leukosis virus strain promotes cell proliferation by negatively regulating 14-3-3σ expressions in chicken fibroblast cells. Viruses.

[32] Mbonye U., Wang B., Gokulrangan G., Shi W., Yang S., Karn J. (2018). Cyclin-dependent kinase 7 (CDK7)-mediated phosphorylation of the CDK9 activation loop promotes P-TEFb assembly with Tat and proviral HIV reactivation. J. Biol. Chem..

[33] Mbonye U.R., Gokulrangan G., Datt M., Dobrowolski C., Cooper M., Chance M.R. (2013). Phosphorylation of CDK9 at Ser175 enhances HIV transcription and is a marker of activated P-TEFb in CD4(+) T lymphocytes. PLoS Pathog..

[34] Pak V., Eifler T.T., Jäger S., Krogan N.J., Fujinaga K., Peterlin B.M. (2015). CDK11 in TREX/THOC regulates HIV mRNA 3' end processing. Cell Host Microbe..

[35] Tsuji K., Mizumoto K., Yamochi T., Nishimoto I., Matsuoka M. (2002). Differential effect of Ik3-1/cables on p53- and p73-induced cell death. J. Biol. Chem..

[36] Wu C.L., Kirley S.D., Xiao H., Chuang Y., Chung D.C., Zukerberg L.R. (2001). Cables enhances cdk2 tyrosine 15 phosphorylation by Wee1, inhibits cell growth, and is lost in many human colon and squamous cancers. Cancer Res..

[37] Shi Z., Li Z., Li Z.J., Cheng K., Du Y., Fu H. (2015). Cables1 controls p21/Cip1 protein stability by antagonizing proteasome subunit alpha type 3. Oncogene.

[38] Wu H., Shi L., Zhang Y., Peng X., Zheng T., Li Y. (2019). Ubiquitination is essential for avibirnavirus replication by supporting VP1 polymerase activity. J. Virol..

[39] Liao T.L., Wu C.Y., Su W.C., Jeng K.S., Lai M.M. (2010). Ubiquitination and deubiquitination of NP protein regulates influenza A virus RNA replication. EMBO J..

[40] Garrus J.E., von Schwedler U.K., Pornillos O.W., Morham S.G., Zavitz K.H., Wang H.E. (2001). Tsg101 and the vacuolar protein sorting pathway are essential for HIV-1 budding. Cell.

[41] Demirov D.G., Ono A., Orenstein J.M., Freed E.O. (2002). Overexpression of the N-terminal domain of TSG101 inhibits HIV-1 budding by blocking late domain function. Proc. Natl. Acad. Sci. U. S. A..

[42] Vana M.L., Tang Y., Chen A., Medina G., Carter C., Leis J. (2004). Role of Nedd4 and ubiquitination of Rous sarcoma virus Gag in budding of virus-like particles from cells. J. Virol..

[43] Patnaik A., Chau V., Wills J.W. (2000). Ubiquitin is part of the retrovirus budding machinery. Proc. Natl. Acad. Sci. U. S. A..

[44] Guan X., Zhang Y., Liu Y., Qi X., Wang Y., Liu C. (2017). Rescue of recombinant RCAS(J)-Luciferase virus and its application in the study of subgroup J avian leukosis virus receptor. Chin J. Prevent. Vet. Med..

[45] Qin L., Gao Y., Ni W., Sun M., Wang Y., Yin C. (2013). Development and application of real-time PCR for detection of subgroup J avian leukosis virus. J. Clin. Microbiol..

[46] Islam A., Cheetham B.F., Mahony T.J., Young P.L., Walkden-Brown S.W. (2006). Absolute quantitation of Marek's disease virus and Herpesvirus of turkeys in chicken lymphocyte, feather tip and dust samples using real-time PCR. J. Virol. Methods.

[47] Yu M., Zhang Y., Zhang L., Wang S., Liu Y., Xu Z. (2024). N123I mutation in the ALV-J receptor-binding domain region enhances viral replication ability by increasing the binding affinity with chNHE1. PLoS Pathog..

